# The inhibitory effect of *Hypericum japonicum* on H9N2 avian influenza virus

**DOI:** 10.1007/s44307-024-00046-4

**Published:** 2024-11-06

**Authors:** Huiqiong Hu, Jingmin Li, Shumei Zheng, Keyu Zhao, Yongbo Xia, Xiaona Wei, Mingzheng Han, Yukun Zhao, Ouyang Peng, Zhiqing Zhao, Zexin Chen, Weiwei Su, Yongchang Cao, Yonggang Wang, Chunyi Xue

**Affiliations:** 1 State Key Laboratory of Biocontrol, School of Life Sciences, Sun Yat-Sen University, Guangzhou, China; 2 School of Life Sciences, Zhengzhou University, Zhengzhou, Henan China; 3 College of Animal Science, South China Agricultural University, Guangzhou, China

**Keywords:** H9N2 avian influenza virus, Antiviral effect, *Hypericum japonicum*, Chickens

## Abstract

The H9N2 subtype of avian influenza virus (AIV) causes severe immunosuppression and high mortality in view of its frequent co-infection with other pathogens, resulting in significant economic losses in the poultry industry. Current vaccines provide suboptimal immune protection against H9N2 AIV owing to antigenic variations, highlighting the urgent need for safe and effective antiviral drugs for the prevention and treatment of this virus. This study aimed to investigate the inhibitory effects of *Hypericum japonicum* extract on H9N2 AIV. Our findings revealed that the extract obtained through resin column separation using 60% ethanol (S06-60%) inhibited H9N2 AIV replication in Madin-Daby canine kidney cells in a dose-dependent manner. The maximum safe concentration of the water-soluble S06-60% extract was determined to be 0.05 mg/mL. Time-course experiments indicated that S06-60% primarily exerted its antiviral effects during the viral pretreatment and adsorption stages. Furthermore, in vivo experiments conducted on specific pathogen-free chickens confirmed the effectiveness of S06-60% in inhibiting H9N2 AIV infection and mitigating associated damage to tracheal and lung tissues. Overall, our study highlights the therapeutic potential of *Hypericum japonicum* extract S06-60% as a viable antiviral candidate against H9N2 AIV, offering promising implications for its application in poultry health management to reduce the economic impact on the poultry industry.

**Supplementary Information:**

The online version contains supplementary material available at 10.1007/s44307-024-00046-4.

## Introduction

Discovered in China in 1994, the H9N2 subtype of avian influenza virus (AIV) has become a significant pathogen threatening the poultry industry (Chen 1997). As a member of the influenza A virus family, H9N2 AIV is an enveloped virus with single-stranded, negative-sense RNA composed of eight segments encoding ten viral proteins (Ghoke et al. 2018; Samji 2009; Wang et al. 2021a, b). Clinical signs of H9N2 outbreaks in chickens are typically mild and include lethargy, reduced appetite, decreased egg production in layers, and slower growth in broilers (Gu et al. 2008). However, H9N2 infections frequently lead to secondary infections by pathogens such as *Escherichia coli*, Newcastle disease virus, infectious bronchitis virus, and other respiratory pathogens, resulting in severe respiratory symptoms, diarrhea, and visceral inflammation, including hepatitis, pericarditis, and peritonitis (Barbour et al. 2009; Monto et al. 2000). Co-infections worsen symptoms, significantly increase mortality rates, and cause substantial economic losses in the poultry industry (Erfan et al. 2019).

Inactivated vaccines have been developed using existing viruses to effectively prevent H9N2 infections. Although these vaccines play a crucial role in preventing and controlling H9N2 epidemics, their efficacy is limited by the ongoing evolution and antigenic drift of the virus (Xu et al. 2018). In this context, traditional Chinese medicines, known for their abundant resources and low potential for the development of resistance, offer a promising alternative for the development of anti-H9N2 AIV drugs. Previous studies have demonstrated the antiviral activity of various medicinal plant components, including isatis root, andrographolide and its derivatives, aqueous extracts of *Radix ilicis asprellae*, and hypericin, against H9N2 AIV (Pourghanbari et al. 2016; Wei et al. 2020). However, only a few effective traditional Chinese antiviral medicines have been widely adopted in poultry production so far.

In recent years, awareness of the antiviral properties of plant extracts and their derivatives has grown (Fang et al. 2018; Sharma and Gupta 2015). With over 40% of modern medicines derived from plants, plant-based compounds may offer an effective approach to controlling H9N2 AIV in chickens (Rajasekaran et al. 2013). *Hypericum japonicum*, also known as *Herba hyperici japonici* from the *Hypericum* species, was first mentioned in the 1848 *Illustrated Catalogue of Plants* (Lin et al. 2004). Extracts from *Hypericum japonicum* have shown inhibitory effects against porcine epidemic diarrhea virus (PEDV) both in vitro and in vivo (Rao et al. 2023). Polyterpenoids isolated from *Hypericum japonicum* have demonstrated anti-herpesvirus activity (Wu et al. 2018a), and newly identified α-pyrones from the plant exhibit potential inhibitory effects on the cleavage and replication of Kaposi’s sarcoma-associated herpesvirus (Hu et al. 2016). In addition, oral administration of *Hypericum japonicum* significantly reduced inflammatory lung lesions caused by H3N2 influenza virus infection and decreased mortality in infected mice (Liu et al. 2008). *Hypericum japonicum* extract also effectively inhibited the proliferation of duck hepatitis B virus, reducing aspartate aminotransferase and alanine transaminase activity as well as viral surface antigen levels (Li et al. 2011). Despite these proven antiviral properties, the efficacy of *Hypericum japonicum* and its derivatives against H9N2 AIV remains unknown. Therefore, this study aimed to investigate the potential inhibitory effects of *Hypericum japonicum* extract on H9N2 AIV in vitro and in vivo. The findings suggest that *Hypericum japonicum* could be applied to prevent and treat H9N2 AIV infections.

## Materials and methods

### Cells and viruses

Madin-Daby canine kidney (MDCK) cells, generously provided by Wen’s Foodstuffs Group Co., Ltd (Guangdong, China), were cultured in Dulbecco’s modified Eagle’s medium (DMEM) (Hyclone, USA) supplemented with 10% fetal bovine serum (FBS) (Thermo Scientific), penicillin (100 U/mL), and streptomycin (100 U/mL) in 5% CO_2_ at 37 ℃.

H9N2 strains (A/Chicken/Guangdong/69/2009) (GenBank accession no. KF514116.1) were isolated from Guangdong Province, China. Virus propagation followed a previously described method with some modifications (Zhang et al. 2017). Briefly, the viruses were propagated in 10-day-old specific pathogen-free (SPF) embryonated chicken eggs. After 72 h, the allantoic fluids were collected and stored at -80 ℃. Viral titers were determined using the Reed-Muench method (Table S1) (Reed and Muench 1938) and a hemagglutination assay (Fig. S1). The cells were then seeded into T75 flasks and incubated at 37 ℃ until they reached 90% confluency. After discarding the medium, 10 mL of DMEM, 10 μL of propagated allantoic fluid, and 10 μL of N-tosyl-L-phenylalanine chloromethyl ketone-treated trypsin (TPCK-trypsin; 1 μg/mL) (Sigma, USA) were added to each flask. After 2 h of incubation, the liquid was discarded, and 10 mL of maintenance medium, consisting of DMEM supplemented with TPCK-trypsin (1 μg/mL), was added. The flasks were incubated at 37 ℃ for 48–72 h until the cytopathic effect (CPE) was observed in more than 90% of the cell monolayer. The flasks were subsequently frozen at -80 ℃ and thawed twice to harvest the virus. Viral titers were determined using the Reed-Muench method (Reed and Muench 1938) and expressed as 50% tissue culture infective dose (TCID_50_).

### Preparation of *Hypericum japonicum* extracts

The traditional Chinese medicine “Tianjihuang” (specimen number: WZ01) was purchased from Yulin, China, and identified as the dried whole herb of *Hypericum japonicum* by senior pharmacist Wei Peng. The plant material was stored at the Guangzhou Quality Research and Development Center of Traditional Chinese Medicine, Sun Yat-sen University, Guangzhou, China. Dried whole herbs (500 g) of *Hypericum japonicum* were extracted using a traditional Chinese extracting machine (PYJ-13, Wenzhou Dingli Medical Equipment Co., Ltd, Wenzhou, China) under optimal conditions: a material-to-water ratio of 1:12 and an extraction time of 30 min. The extraction process was repeated three times. The resulting aqueous extract was filtered through a 400-mesh filter cloth and purified using D101 macroporous resin (Zhengzhou Hecheng New Materials Technology Co., Ltd).

The optimal purification process involved packing the resin column with a medicinal herb-to-resin ratio of 1:2. The extract was applied to the column, which was initially eluted with 6 bed volumes (BV) of water to remove impurities, followed by elution with 4 BV of 20% ethanol and 4 BV of 60% ethanol. Each eluent was concentrated using a rotary evaporator (R-200, BUCHI) and freeze-dried (TFD0.6, Guangdong Zhensheng Intelligent Equipment Co., Ltd) to yield three fractions: 44.30 g from the water elution (S06-0%), 8.96 g from the 20% ethanol elution (S06-20%), and 23.70 g from the 60% ethanol elution (S06-60%).

One hundred grams (100 g) of dried whole herb of *Hypericum japonicum* was extracted with water following the method described above, yielding 16.03 g of residue (S06-0) after concentration and freeze-drying. Ethanol extraction of the same amount of dried herb produced 15.05 g of crude extract (S06-CE). Oseltamivir was purchased from Roche Pharmaceutical Company.

To prepare the concentration liquid, 20 mg of each extract (S06-CE, S06-0, and S06-0%) was dissolved in 10 mL of sterile water, resulting in solutions with a concentration of 2 mg/mL. S06-20% and S06-60% were prepared using sterile water and 50% absolute ethanol, respectively. The oseltamivir solution was prepared by dissolving 4 mg of oseltamivir in 0.1 mL of dimethyl sulfoxide (DMSO), followed by 100-fold dilution in DMSO to reach a final concentration of 40 μg/mL. All solutions were sterilized by filtration through a 0.22 μm pore size filter and stored at 4 ℃.

### CCK-8 assay

The cytotoxicity of *Hypericum japonicum* extracts (S06-CE, S06-0, S06-0%, S06-20%, and S06-60%) and oseltamivir in MDCK cells was evaluated using the Cell Counting Kit-8 (CCK-8; Yeasen Biotech, China) assay. Briefly, MDCK cells were seeded into 96-well plates and incubated at 37 ℃ until they reached 90% confluency. The extracts and oseltamivir were diluted with DMEM to various concentrations and added to the 96-well plates, along with control DMEM or DMEM containing 0.1% solvent (DMSO, sterile water, or 50% ethanol). After 24 and 48 h, 10% CCK-8 solution in DMEM was added to the plates. The plates were then incubated in the dark for 1 h, after which optical density (OD) values were measured at 450 nm. The relative viability of the cells was calculated using the following formula: cell survival rate (%) = [(OD_sample_ – OD_blank_) / (OD_control_ – OD_blank_)] × 100.

### Inhibition of viral infection: in vitro assay

MDCK cells were seeded into 12-well plates and incubated until they reached 90% confluency. The cells were then treated for 1 h with various concentrations of *Hypericum japonicum* extracts (S06-CE, S06-0, S06-0%, S06-20%, and S06-60%) and oseltamivir, control DMEM, or DMEM with 0.1% solvent. After incubation, the cells were infected with H9N2 AIV at a titer of 2.15 $\times$ 10^7^ TCID_50_ for 2 h. The viral inoculum was subsequently removed, and fresh maintenance medium containing TPCK-trypsin (1 μg/mL), along with different concentrations of the extracts and oseltamivir, was added. After 24 h of incubation, indirect immunofluorescence analysis (IFA) was performed. The expression of nucleocapsid (N) and glyceraldehyde 3-phosphate dehydrogenase (GAPDH) proteins was assessed using western blotting, and the relative transcription levels of matrix (M) genes were evaluated using quantitative reverse transcription polymerase chain reaction (RT-qPCR). Viral titers were determined by TCID_50_ analysis.

### Immunofluorescence assay

IFA was performed on MDCK cells infected with H9N2 AIV, following a previously described method with minor modifications (Xu et al. 2020). Briefly, the cells were washed three times with phosphate-buffered saline (PBS) with Tween 20 (PBST) and fixed in 4% pre-cooled paraformaldehyde at 4 ℃ for 15 min. The cells were then permeabilized with 0.5% Triton X-100 for 15 min and blocked with 3% bovine serum albumin (BSA) at room temperature for 1 h or overnight at 4 ℃. Subsequently, the cells were incubated with anti-H9N2 AIV N monoclonal antibody (Abcam) at a 1:1000 dilution in 1% BSA at 37 ℃ for 1 h or overnight at 4 ℃. Following primary antibody incubation, the cells were incubated with fluorescein isothiocyanate-conjugated secondary antibody at a 1:500 dilution in 1% BSA at 37 ℃ for 1 h in the dark. To visualize the cell nuclei, the cells were stained with 4’,6-diamidino-2-phenylindole (Genview, Beijing, China) at a 1:20 dilution in PBST for 15 min. After three washes with PBST, viral N protein expression was observed under a fluorescence microscope (LEICA DMi8, Germany).

### Western blot analysis

Proteins were extracted by treating the cells with lysis buffer (Beyotime, China) containing 1% protease inhibitor (Yatai Hengxin, China) on ice for 30 min, followed by centrifugation. The supernatant was then boiled in 5 × sodium dodecyl sulfate (SDS) loading buffer for 5 min. Equal volumes of protein samples were separated using 12.5% SDS–polyacrylamide gel electrophoresis (SDS-PAGE) and transferred onto polyvinylidene fluoride (PVDF) membranes (Millipore, USA). The membranes were blocked with 4% non-fat milk, then incubated overnight at 4 ℃ with either anti-H9N2 AIV N monoclonal antibody (1:500 dilution) (Abcam) or anti-GAPDH antibody (1:1000 dilution) (Proteintech Group, Inc., USA). Afterward, the membranes were incubated with horseradish peroxidase-conjugated goat anti-mouse IgG (1:5000 dilution) (Proteintech, USA) at room temperature for 1 h. The chemiluminescent signals emitted from the protein blots were visualized using enhanced chemiluminescence (ESL) reagents (NMC Biotech) and captured with a GelView 6000 Pro imaging system following the manufacturer’s instructions (BLT, Guangdong, China).

### Time-of-addition assay

MDCK cells were cultured overnight in 12-well plates before being infected with H9N2 AIV at a titer of 2.15 $\times$ 10^7^ TCID_50_. The plates were incubated at 4 ℃ for 1 h to allow for virus adsorption. After removing the inoculum, each well was supplemented with DMEM containing TPCK-trypsin (1 μg/mL), and the plates were placed at 37 ℃. S06-60% was added at a concentration of 0.05 mg/mL at various time points (Fig. 4A). After 12 h, the expression levels of viral N protein were determined by western blot analysis, and viral titers were assessed using the TCID_50_ assay. The relative transcription levels of the viral M gene were determined using RT-qPCR.

### RNA extraction, RT-PCR analysis, and RT-qPCR analysis

Total viral RNA was extracted from throat and cloacal swabs collected from the animals using a viral nucleic acid extraction kit (Magen, Guangzhou, China). The extracted RNA was then subjected to complementary DNA (cDNA) synthesis using a RT-PCR kit (TaKaRa, Dalian, China). PCR was performed using primers specific for the H9N2 AIV hemagglutinin (HA) gene. The sense primer sequence was 5’-CCAGCAAAAGCAGGGGAATTTC-ACA-3’, and the antisense primer sequence was 5’-TTAGTAGAAACAAGGGTGTTTTTGCCAA-3’. The PCR reactions were carried out in 20 μL volumes, which included 0.8 μL of PrimeScript 1 Step Enzyme Mix, 10 μL of 2 × 1 Step Buffer, 0.8 μL of the HA gene-specific primers, and 8.4 μL of template RNA. The reaction conditions were as follows: 50 ℃ for 30 min; 94 ℃ for 2 min; 30 cycles of 94 ℃ for 30 s, 55 ℃ for 30 s, and 72 ℃ for 100 s; followed by a final step at 4 ℃. After preparing a 1% nucleic acid gel, PCR samples were loaded and electrophoresed at 190 V for 30 min.

For RT-qPCR, viral RNA was extracted from MDCK cells, and swabs were collected from the animals. The RNA was used for cDNA synthesis with a one-step transcription kit (TaKaRa, Dalian, China). qPCR was performed using primers specific for the H9N2 AIV M gene, with GAPDH as the internal reference gene. The sense primer sequence for the H9N2 AIV M gene was 5’-CTTCTAACCGAGGTCGAAACG-3’, and the antisense primer sequence was 5’-AGGGCATTTTGGACAAAKCGTCTA-3’. The sense primer sequence for GAPDH was 5’-TGCCATCACAGCCACACAGAAG-3’, and the antisense primer sequence was 5’-ACTTTCCCCACAGCCTTAGCAG-3’. The reactions were carried out in 10 μL volumes, which included 5 μL of 2 × Color SYBR Green qPCR Master Mix (ROX2 plus), 0.4 μL of each gene-specific primer, and 1 μL of cRNA. qPCR was performed using a LightCycler 480 instrument (Roche, Basel, Switzerland). The reaction conditions were as follows: 95 ℃ for 5 min; 40 cycles of 95 ℃ for 10 s and 60 ℃ for 30 s; followed by a final step at 4 ℃. To confirm the specificity of the amplification, melt curve analysis was performed with the following conditions: 95 ℃ for 15 s, 60 ℃ for 1 min, and 95 ℃ for 30 s. The messenger RNA (mRNA) transcription level of the H9N2 AIV M gene was quantified using the cycle threshold (Ct) value, normalized to that of GAPDH.

### Animal experiments

The aim of this study was to investigate the effects of S06-60% on SPF chickens infected with H9N2 AIV. The chickens were housed in individual isolators under positive pressure. Eighty 22-day-old SPF chickens were randomly divided into four groups. The S06-60% group received 1 mL of S06-60% orally for 10 consecutive days at a dosage of 1.4 g/kg of body weight. The positive control group was orally administered oseltamivir at a dosage of 20 mg/kg. At 32 days of age, all groups except the negative control group were intranasally inoculated with 200 μL of H9N2 AIV (A/Chicken/Guangdong/69/2009 (H9N2)) at a titer of 10^8.5^ 50% egg infectious dose (EID_50_), whereas the negative control group received 200 μL of 1 × PBS buffer instead. Body weight was recorded every two days. Blood samples were collected from the S06-60% and negative control groups one day before the viral challenge for enzyme-linked immunosorbent assay (ELISA) analysis. Throat and cloacal swabs were collected from five chickens in each group at 1, 2, 3, and 4 days post-infection (dpi) for RT-PCR and RT-qPCR analyses. Blood samples were also collected at these time points, and serum was separated for interferon gamma (IFN-γ) and interleukin-6 (IL-6) detection using ELISA. At the end of the experiment (15 dpi), lung and tracheal samples were collected for hematoxylin and eosin (H&E) staining and microscopic observation.

#### Enzyme-linked immunosorbent assay

The levels of cytokines (IFN-γ and IL-6) in serum samples were determined using chicken ELISA kits (CUSABIO, Wuhan, China) according to the instructions of the manufacturer. Briefly, samples were added to the ELISA plate, followed by the working solution, and then incubated at 37 ℃ for 30 min. After washing, substrate solution was added, and the plate was incubated at 37 ℃ for 20 min. The reaction was halted with a stop solution, and the OD value of each well was measured at 450 nm using a microplate reader to determine cytokine concentrations by interpolation of the standard curve based on the OD values.

#### Histopathology

In the animal experiments, lung and tracheal tissues from SPF chickens were collected post-dissection and fixed in 10% formaldehyde at room temperature. The tissues underwent standard processing for histological analysis, which included dehydration, clearing, and embedding in paraffin wax. Tissue sections were then mounted on glass slides, stained with H&E, and observed under a light microscope.

#### Statistical analysis

All data were analyzed using GraphPad Prism 8 software and are presented as mean ± standard deviation. Statistical significance between groups was determined using the t-test, with *P* < 0.05 considered significant. 

## Results

### Evaluation of the cytotoxicity of *Hypericum japonicum* extracts

**Fig. 1:**
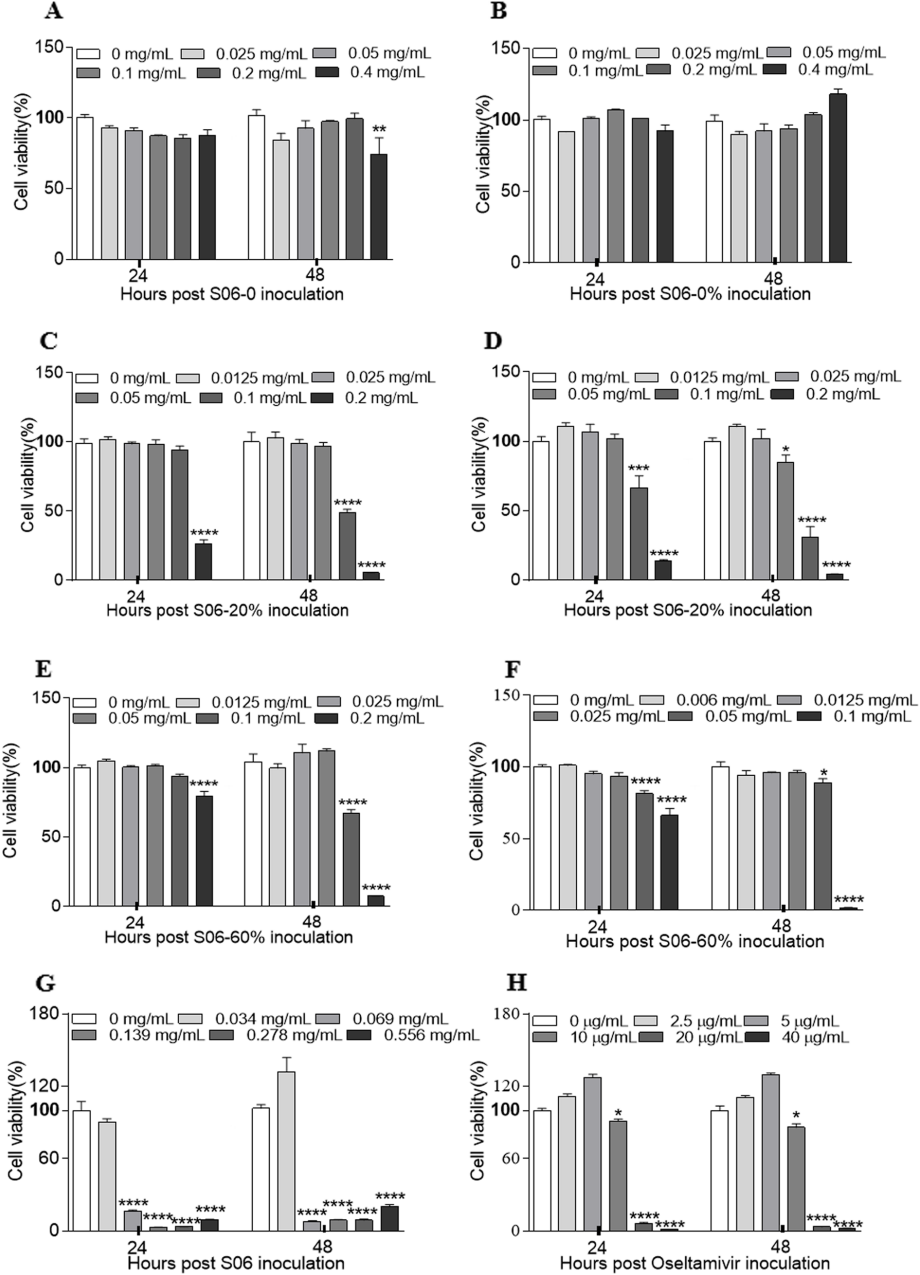
Viability of MDCK cells in presence of the *Hypericum japonicum* extracts with different concentrations. MDCK cells were cultured with or without various concentrations of the extracts, for 24 h and 48 h prior to the CCK-8 assay. **A**: S06-0; **B**: S06-0%; **C**: S06-20% dissolved in water; **D**: S06-20% dissolved in 50% ethanol; **E**: S06-60% dissolved in water; **F**: S06-60% dissolved in 50% ethanol; **G**: S06-CE; **H**: Oseltamivir (W: The extract was dissolved in sterile water; A: The extract was dissolved in 50% ethanol). Data are represented as mean ± SD, *n* = 8. * represents *P* < 0.05; ** represents *P* < 0.01; *** represents *P* < 0.001; **** represents *P* < 0.0001

To assess the cytotoxic potential of the *Hypericum japonicum* extracts, the viability of MDCK cells was evaluated using the CCK-8 assay. The cells were exposed to various concentrations of S06-CE, S06-0, S06-0%, S06-20%, S06-60%, and oseltamivir for 24 and 48 h. The OD at 450 nm was measured in each well of a 96-well plate using a microplate reader. As shown in Fig. 1, the maximum safe concentrations for MDCK cells were determined as follows: S06-0—0.2 mg/mL; S06-0%—0.4 mg/mL; S06-20% (aqueous solution)—0.05 mg/mL; S06-20% (dissolved in 50% ethanol)—0.025 mg/mL; S06-60% (aqueous solution)—0.05 mg/mL; S06-60% (dissolved in 50% ethanol)—0.025 mg/mL; S06-CE—0.034 mg/mL; and oseltamivir—5 μg/mL.

### *Hypericum japonicum* inhibits H9N2 AIV infection in vitro

**Fig. 2:**
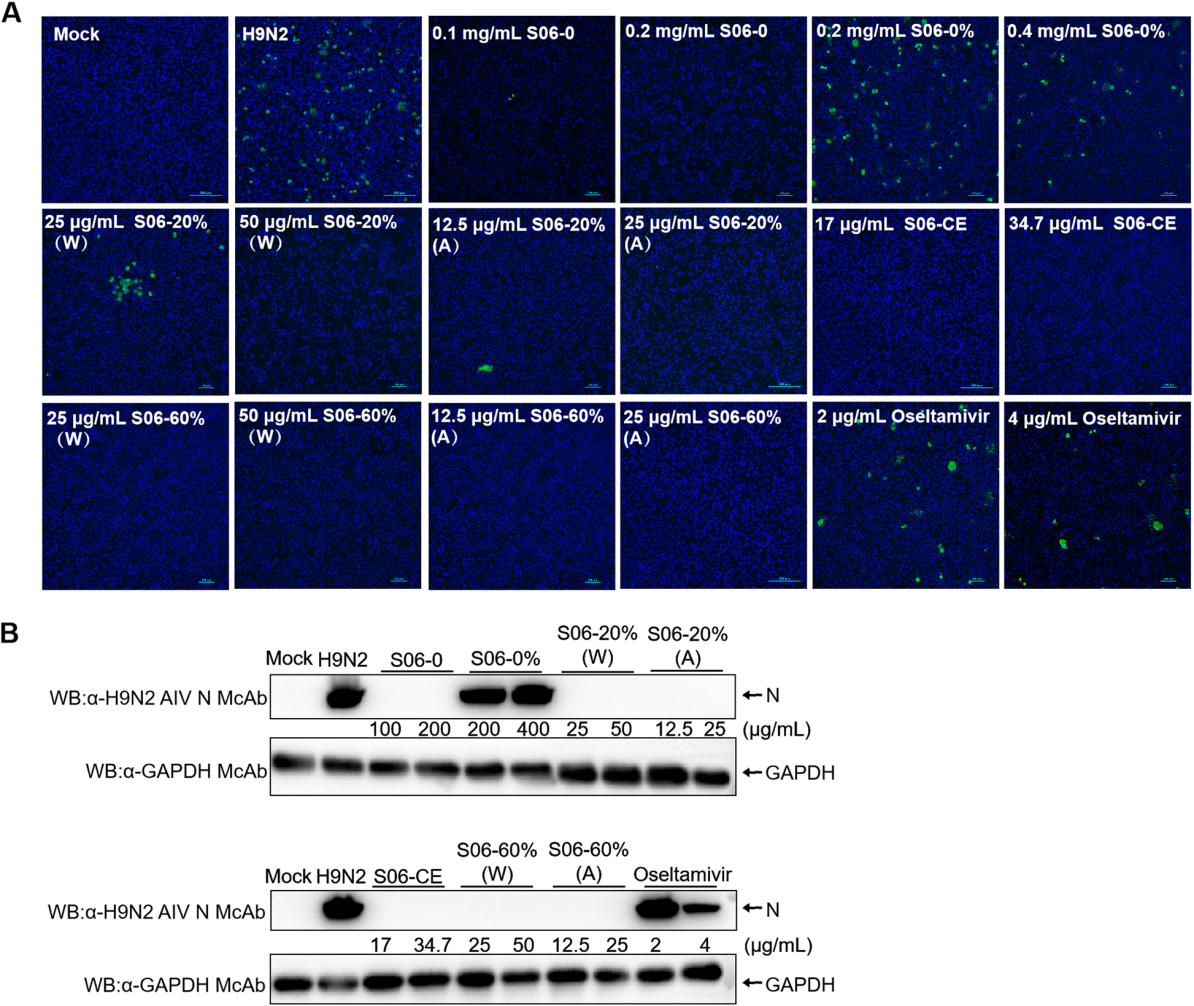
S06-60% inhibits H9N2 AIV replication in vitro. MDCK cells were treated with or without various concentrations of the extracts for 1 h, followed by infection with H9N2 AIV (1:1000). After 2 h, the cells were re-treated with the extract or DMEM as control. (**A**) At 24 h post-inoculation, an indirect immunofluorescence assay was performed. CPE and H9N2 AIV antigen were indicated by green fluorescence. (**B**) MDCK cells were treated as described above. After 24 h, cell lysates were prepared and examined with Western Blot using anti-H9N2 AIV N polyclonal antibody. (W: The extract was dissolved in sterile water; A: The extract was dissolved in 50% ethanol). Data are represented as mean ± SD, *n* = 3

To examine the effect of *Hypericum japonicum* on H9N2 infection, MDCK cells were treated with *Hypericum japonicum* extracts at various concentrations, based on the maximum safe concentrations determined in the previous cell viability assay. The antiviral activity of these extracts against H9N2 AIV was subsequently evaluated using IFA and western blot analysis. Figure 2A shows the results of the IFA, indicating that treatment with S06-0, S06-20% (dissolved in water or 50% ethanol), S06-60% (dissolved in water or 50% ethanol), S06-CE, and oseltamivir significantly reduced the expression of the H9N2 AIV N protein after 24 h of incubation. Among these, S06-60% (dissolved in water or 50% ethanol) exhibited the most pronounced inhibitory effects. However, treatment with S06-0% did not result in a significant change in H9N2 AIV N protein expression.

To further validate the anti-H9N2 AIV effect of the extracts, western blot analysis was performed using a monoclonal antibody against the N protein. Figure 2B demonstrates that the H9N2 AIV N protein could not be detected after treatment with S06-0, S06-20% (dissolved in water or 50% ethanol), S06-60% (dissolved in water or 50% ethanol), or S06-CE. Oseltamivir at 4 μg/mL significantly reduced the expression of the H9N2 AIV N protein. Conversely, treatment with S06-0% did not induce a significant change in H9N2 AIV N protein expression, which was consistent with the IFA results. Considering the higher maximum safe concentration and safety concerns in animal experiments, S06-60% (aqueous solution) was chosen as the primary focus for further investigation instead of S06-CE, which is a crude extract.

### S06-60% dose-dependently inhibits H9N2 AIV infection in vitro

**Fig. 3:**
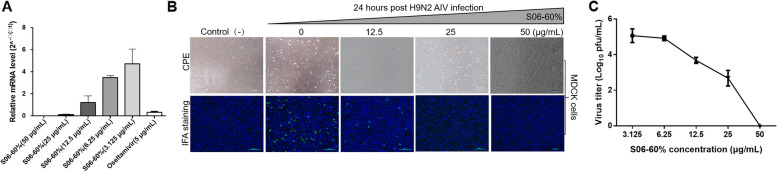
S06-60% dose-dependently inhibits H9N2 AIV replication in vitro. MDCK cells were treated with DMEM as a control medium or with various concentrations of S06-60% for 1 h, followed by infection with H9N2 AIV (1:1000). After 2 h, the cells were re-treated with S06-60% of DMEM as a control. (**A**) At 24 h post-inoculation, an indirect immunofluorescence assay was performed to detect CPE and H9N2 AIV antigen, which are indicated by green fluorescence. (**B**) MDCK cells were treated as described above. After 24 h, cell lysates were prepared and examined using Western Blot with anti-H9N2 AIV N polyclonal antibody. (**C**) Virus titers in the cell lysates were determined by TCID_50_ analysis. The data are represented as mean ± SD, with *n* = 3 replicates

The effect of S06-60% (aqueous solution) on H9N2 AIV infection in MDCK cells was further explored by analyzing M gene mRNA transcription, N protein expression, and viral titers. Figure 3A shows that lower concentrations of S06-60% resulted in higher levels of M gene mRNA transcription, demonstrating dose-dependent inhibition of H9N2 AIV. Specifically, S06-60% at 50 μg/mL and 25 μg/mL effectively suppressed M gene mRNA transcription (*P* < 0.0001). Figure 3B illustrates the fact that decreasing S06-60% concentrations correlated with increased N protein expression, with minimal detection at 50 μg/mL and 25 μg/mL. Furthermore, viral titer analysis revealed a reduction in viral particles at higher concentrations of S06-60%, particularly with minimal detection at 50 μg/mL (Fig. 3C). Overall, these results indicate that the inhibitory effect of S06-60% on H9N2 AIV in MDCK cells is dose-dependent, with optimal inhibition observed at concentrations of 50 μg/mL and 25 μg/mL.

### S06-60% primarily exerts its antiviral effect on H9N2 AIV during the pretreatment and adsorption stages of the viral replication cycle

**Fig. 4:**
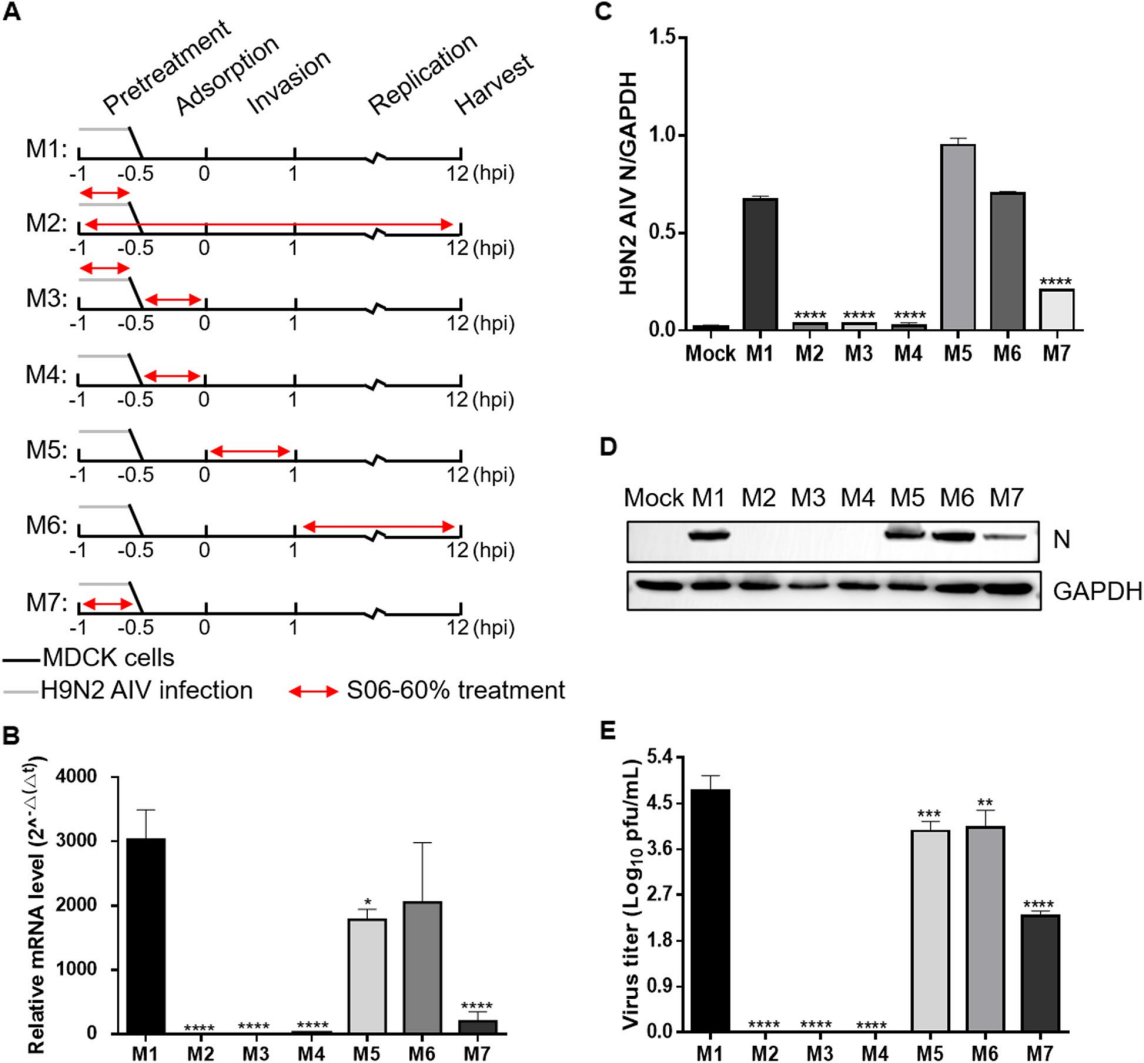
Time-of-addition experiments. (**A**) MDCK cells infected with H9N2 AIV were treated with S06-60% at various time points before and after infection. The presence of S06-60% is indicated by double-headed red arrows. The experiments are identified in the text by the numbers on the left (M1-M7). Cells were harvested at 12 hpi. (**B**) The mRNA expression of M was examined with real-time PCR using specific primers. The expression level of mRNA was calculated in relation to the expression level of GAPDH. (**C**) The gray-scale scanning ratio of N protein-specific bands to GAPDH-specific bands was determined from Western Blot results; (**D**) The N protein of H9N2 AIV was detected using anti-H9N2 AIV N polyclonal antibody in Western Blot. (**E**) The virus titers in the culture medium were determined by TCID_50_ analysis. The data are represented as mean ± SD with *n* = 3 replicates. * represents *P* < 0.05; ** represents *P* < 0.01; *** represents *P* < 0.001; **** represents *P* < 0.0001

An experiment was conducted to determine the specific stage at which S06-60% exerts its antiviral effect by treating H9N2 AIV at different stages of infection. As illustrated in Fig. 4A, the experiment comprised seven groups (M1-M7) (Fang et al. 2018; Sharma and Gupta 2015). M1 was the control group, in which no extract was added during the viral replication stage. M2 involved the addition of S06-60% throughout all stages of H9N2 AIV infection. M3 entailed premixing S06-60% with H9N2 AIV. M4 involved adding S06-60% during the H9N2 AIV adsorption stage. M5 included the addition of S06-60% during the H9N2 AIV invasion stage. M6 involved adding S06-60% during H9N2 AIV replication. M7 entailed premixing S06-60% with the cells.

After 12 h of viral infection, RT-qPCR, western blotting, and TCID_50_ assays were conducted to compare the mRNA transcription levels of the H9N2 AIV M gene, N protein expression levels, and viral titers. As depicted in Figs. 4B-E, the M2, M3, M4, and M7 treatment groups showed significant suppression of H9N2 AIV transcription and protein expression, along with decreased viral titers (*P* < 0.0001), compared with the M1 control group. These findings indicate that S06-60% primarily exerts its inhibitory effect during the pretreatment and adsorption stages of H9N2 AIV infection.

### S06-60% inhibits H9N2 AIV infection in SPF chickens

**Fig. 5:**
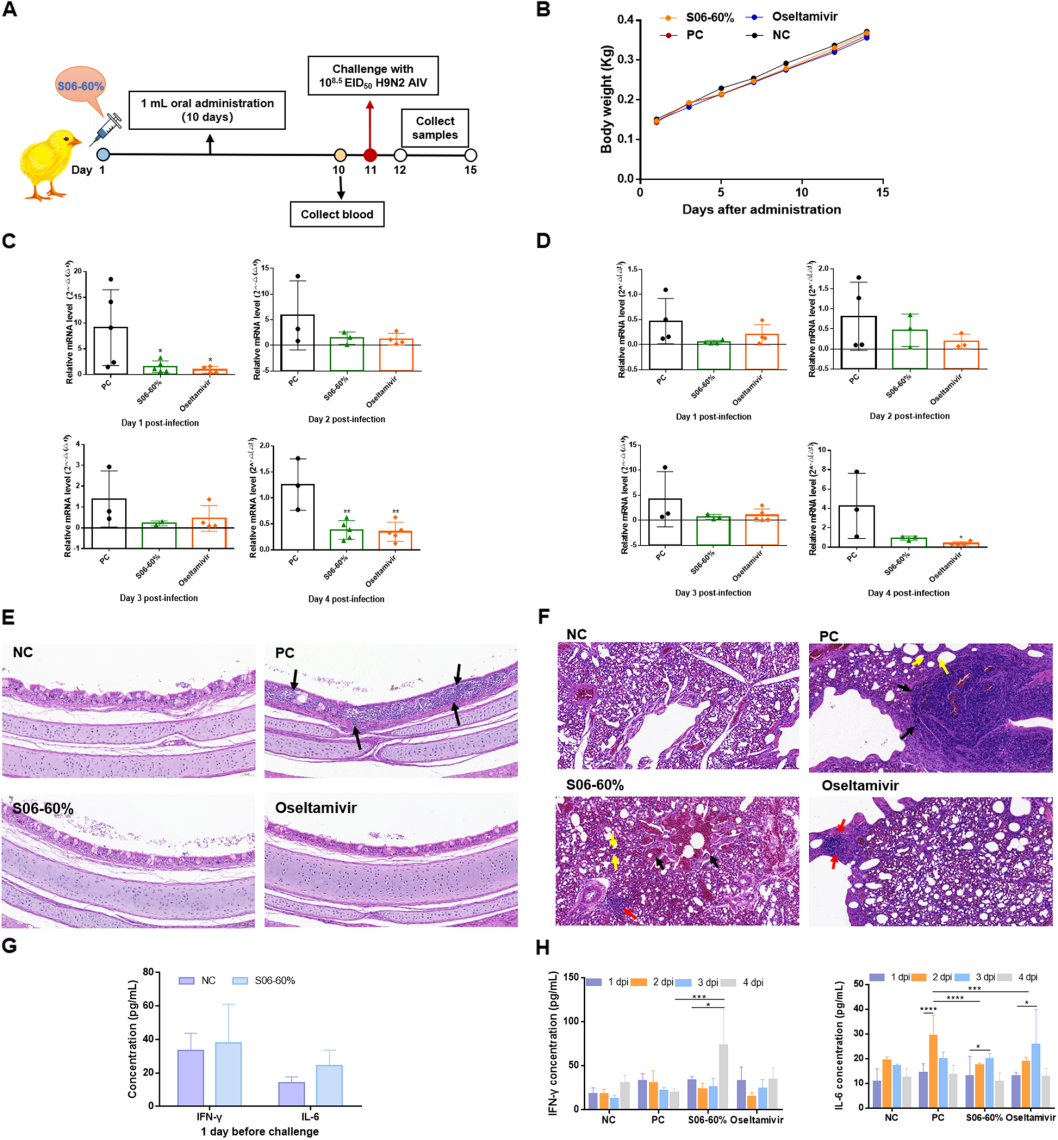
The protective effect of oral administration of S06-60% against H9N2 AIV infection in SPF chickens. (**A**) Experimental diagram: 80 SPF chickens at 22 days of age were randomly divided into 4 groups. The S06-60% group received oral administration of 1 mL of S06-60% per day for 10 consecutive days. At 32 days of age, all groups, except the negative control group (administered 200 μL of 1 × PBS buffer), were challenged with 200 μL of H9N2 AIV at a titer of 10.^8.5^ EID_50_. (**B**) The body weights of SPF chickens were monitored throughout the experiment. (**C**) The H9N2 AIV loads in throat swabs of SPF chickens was quantified using RT-qPCR. (**D**) The H9N2 AIV loads in the cloacal swabs of SPF chickens was quantified using RT-qPCR. (**E**–**F**) H&E-stained trachea and lung tissue sections from SPF chickens at 4 dpi were examined under a microscope. (**G**) Expression levels of IFN-γ and IL-6 were measured by ELISA one day before challenge. (**H**) Expression levels of IFN-γ and IL-6 were measured by ELISA after challenge. The data are presented as mean ± SD with *n* = 5 replicates. * represents *P* < 0.05; *** represents *P* < 0.001; **** represents *P* < 0.0001

The inhibitory effect of S06-60% was investigated in SPF chickens challenged with H9N2 AIV (Fig. 5A). Figure 5B shows a consistent increase in the body weights of the chickens across all groups after the experiment began, indicating no negative impact of S06-60% on chicken weight. RT-PCR analysis of throat swabs collected on day 1 post-challenge (Table 1) revealed no viral bands in the S06-60% group, suggesting that S06-60% reduced early viral levels and demonstrated an inhibitory effect on H9N2 AIV infection.

**Table 1:** Statistics analysis of the RT-PCR results for the presence of H9N2 AIV HA gene in throat swabs collected on the first day after challenge

Group	Positive samples /Total samples
S06-60%	0/5
Oseltamivir	1/5
PC	3/5
NC	0/5

Because no viral bands were detected in throat swabs at 2, 3, and 4 dpi, or in any cloacal swabs, RT-qPCR was employed (Fig. 5C-D). The viral loads in the throat swabs from both the S06-60% and oseltamivir groups were lower than those in the positive control group post-challenge. At 1 dpi, both the S06-60% and oseltamivir groups exhibited significantly reduced viral loads compared with the positive control group (*P* < 0.05), and this difference remained significant at 4 dpi (*P* < 0.01). The results from the cloacal swabs were similar, showing lower viral loads in the S06-60% and oseltamivir groups after the challenge, with significantly reduced viral levels in the oseltamivir group at 4 dpi (*P* < 0.05). These findings indicate that both S06-60% and oseltamivir possess antiviral effects against H9N2 AIV in SPF chickens.

Figures 5E-F illustrate the tracheal and lung damage in chickens infected with H9N2 AIV at 4 dpi. The tracheas of the positive control group exhibited focal infiltration of inflammatory cells in the lamina propria, extending to the epithelium and causing it to thin. In contrast, the S06-60% and oseltamivir groups showed reduced inflammation and less tracheal damage. Lung damage was assessed using previously described criteria (Wang et al. 2021a, b), as detailed in Table 2, with the grading results presented in Table 3. In the lungs of the positive control group, lymphoid infiltration was observed around the secondary bronchial mucosa, leading to thickened airways and dilated blood vessels. Conversely, the aforementioned treatment groups exhibited less lung damage and fewer symptoms, such as hemorrhage, inflammation, and airway thickening.

**Table 2:** The criteria for microscopic observation of the lung and trachea

Grade	Microscopic observation
-	It indicates that the organizational structure is normal and there is no significant change
+	It indicates occasional histitis cells
+ +	It indicates a multi-tissue lymphoid focal infiltration that is visible and is clearly visible

**Table 3:** The effect of S06-60% on histopathological changes in lung at 4 days post H9N2 AIV infection

Group	1	2	3	4	5
S06-60%	+	+ +	+	+	+
Oseltamivir	+	+	+	+	+
PC	+	+ +	+ +	+	+ +
NC	-	-	-	-	-

Figures 5G-H illustrate the use of ELISA to detect IFN-γ and IL-6 levels in different groups before and after the viral challenge. The initial levels of these two cytokines in the S06-60% group were comparable to those in the negative control group. After the challenge, S06-60% significantly elevated IFN-γ levels (*P* < 0.05), surpassing those in the positive control group at 4 dpi (*P* < 0.01). IL-6 levels increased at 2 dpi in both the S06-60% and oseltamivir groups (*P* < 0.05) but remained significantly lower than those in the positive control group (*P* < 0.01), suggesting that S06-60% could reduce inflammation and associated post-infection damage.

In summary, S06-60% was effective against H9N2 AIV in SPF chickens, inhibiting viral growth, increasing serum IFN-γ levels, decreasing IL-6 levels, and mitigating damage to tracheal and lung tissues.

## Discussion

H9N2 AIV has become a major concern in China since its first outbreak, causing substantial negative effects on the poultry industry (Jiang et al. 2012; Liu et al. 2014b; Ma et al. 2018; Shang et al. 2010; Wu et al. 2018b; Zeng et al. 2017; Zhu et al. 2014). Initially, H9N2 AIV leads to mild respiratory symptoms in chickens; however, it can progress to severe respiratory issues, immunosuppression, secondary infections, decreased egg production, and high mortality, resulting in significant economic losses (Choi et al. 2004; Dong et al. 2011; Sun and Liu 2015). Although vaccination remains the primary preventive measure against this virus, the ongoing evolution and mutation of H9N2 AIV undermine the effectiveness of existing vaccines (Sun et al. 2012). Therefore, alternative antiviral strategies are urgently needed. In this study, we report the antiviral properties of *Hypericum japonicum* extract against H9N2 AIV infection, both in vitro and in vivo.

Chinese herbal medicines, such as *Hypericum japonicum*, offer several advantages for drug development, including a broader range of targets, fewer side effects, and reduced drug resistance. Known as “di er cao,” *Hypericum japonicum* has been used for nearly a century to treat viral hepatitis and other infectious diseases (Wang et al. 2008a). It contains various bioactive components, including aliphatic compounds, terpenes, flavonoids, xanthones, lactones, and phloroglucinol derivatives (Gao et al. 2009; Hu et al. 2000; Ishiguro et al. 1994; Liu et al. 2014a; Verma et al. 2012; Wang et al. 2008b; Zhang et al. 2007), which exhibit broad pharmacological effects (Li et al. 2008). *Hypericum japonicum* has demonstrated inhibitory effects against PEDV, herpesviruses, Kaposi’s sarcoma-associated herpesvirus, H3N2 influenza virus, and duck hepatitis B virus, making it a valuable resource for antiviral drug development (Gao et al. 2009; Hu et al. 2000; Ishiguro et al. 1994; Liu et al. 2014a; Verma et al. 2012; Wang et al. 2008b; Zhang et al. 2007).

In the present study, we focused on the efficacy of *Hypericum japonicum* efficacy against H9N2 AIV infection in vitro. After determining the maximum safe concentration of *Hypericum japonicum* extracts for MDCK cells using the CCK-8 assay, we evaluated the anti-H9N2 AIV activity of the extracts at the protein level. The results revealed that S06-60%, obtained through resin column separation using 60% ethanol, exhibited the strongest inhibitory action against H9N2 AIV. Dose-dependent inhibition of H9N2 AIV by S06-60% was observed across various levels, including mRNA transcription, protein synthesis, and viral replication. Notably, at a concentration of 0.05 mg/mL, S06-60% completely inhibited H9N2 AIV growth. Although the exact mechanisms remain unclear, our data indicated that introducing S06-60% during the viral pretreatment and adsorption stages nearly completely inhibited H9N2 AIV transcription and expression, resulting in viral titers in MDCK cells dropping to zero, which suggests an almost complete prevention of viral replication.

Furthermore, in vivo experiments with SPF chickens demonstrated that S06-60% could reduce viral loads in throat swabs, regulate immune responses by increasing IFN-γ levels and decreasing IL-6 levels, and mitigate histopathological damage to respiratory tissues compared with the antiviral drug oseltamivir.

In conclusion, *Hypericum japonicum* extract S06-60% exhibits potent activity against H9N2 AIV, confirmed through both in vitro and in vivo studies, with the most significant effects observed during the viral pretreatment and adsorption stages. However, to advance the application of anti-H9N2 AIV therapeutics, further investigations are necessary to identify the active compounds within *Hypericum japonicum* responsible for its antiviral effects and to elucidate the exact underlying mechanisms. Addressing these issues is crucial for developing a new, safe antiviral medication to control H9N2 AIV in poultry.

## Supplementary Information

Supplementary Material 1.

## Data Availability

The data that support the findings of this study are available in the supplementary files of this article.
