# Supplementary material for: The inhibitory effect of Hypericum japonicum on H9N2 avian influenza virus
Source: Adv Biotechnol (Singap). 2024 Nov 6;2(4):41. doi: 10.1007/s44307-024-00046-4 (PMC11740843; doi:10.1007/s44307-024-00046-4)
Supplement: Supplementary file 1 — Supplementary Material 1. [file 44307_2024_46_MOESM1_ESM.docx]

Supplementary Material

Table S1 The results of EID_50_

| Dilution | Total | Infected | Uninfected | Total infected | Total uninfected | Ration | Infection rate（%） |
| --- | --- | --- | --- | --- | --- | --- | --- |
| 10^-4^ | 5 | 5 | 0 | 25 | 0 | 25/25 | 100 |
| 10^-5^ | 5 | 5 | 0 | 20 | 0 | 20/20 | 100 |
| 10^-6^ | 5 | 5 | 0 | 15 | 0 | 15/15 | 100 |
| 10^-7^ | 5 | 5 | 0 | 10 | 0 | 10/10 | 100 |
| 10^-8^ | 5 | 5 | 0 | 5 | 0 | 5/5 | 100 |
| 10^-9^ | 5 | 0 | 5 | 0 | 5 | 0/5 | 0 |

Figure S1 The hemagglutination assay of H9N2 AIV
